# Sustained *in vitro* interferon-beta release and *in vivo* toxicity of PLGA and PEG-PLGA nanoparticles†

**DOI:** 10.1039/c9ra09928j

**Published:** 2020-04-22

**Authors:** Andrea Fodor-Kardos, Ádám Ferenc Kiss, Katalin Monostory, Tivadar Feczkó

**Affiliations:** Institute of Materials and Environmental Chemistry, Research Centre for Natural Sciences Magyar Tudósok Körútja 2 H-1117 Budapest Hungary tivadar.feczko@gmail.com +36-88-624000 ext. 3508; Research Institute of Biomolecular and Chemical Engineering, University of Pannonia Egyetem u. 10 H-8200 Veszprém Hungary; Institute of Enzymology, Research Centre for Natural Sciences Magyar Tudósok Körútja 2 H-1117 Budapest Hungary

## Abstract

Interferon-beta-1a (IFN-β-1a) can diminish the symptoms of relapsing-remitting multiple sclerosis. Herein, we prepared sustained drug delivery IFN-β-1a-loaded nanoparticles by a double emulsion solvent evaporation method. Bovine serum albumin (BSA) model drug was used to optimize the preparation of nanoparticles composed of four types of poly(lactic-*co*-glycolic acid) (PLGA) polymers and two pegylated PLGA (PEG-PLGA) polymers. *Via* optimization, selected PLGA and PEG-PLGA polymers were able to entrap IFN-β-1a with high encapsulation efficiency (>95%) and low size (145 nm and 163 nm, respectively). *In vitro* release kinetics of BSA and IFN-β showed similar tendency for PLGA and PEG-PLGA nanoparticles, respectively. Although the drug loaded nanoparticles did not show toxicity in hepatocyte cells, mild toxic effects such as pale kidney and pyelectasis were observed in the *in vivo* studies.

## Abstract 2

Interferon-beta-1a (IFN-β-1a) can diminish the symptoms of relapsing-remitting multiple sclerosis.

## Graphical abstract

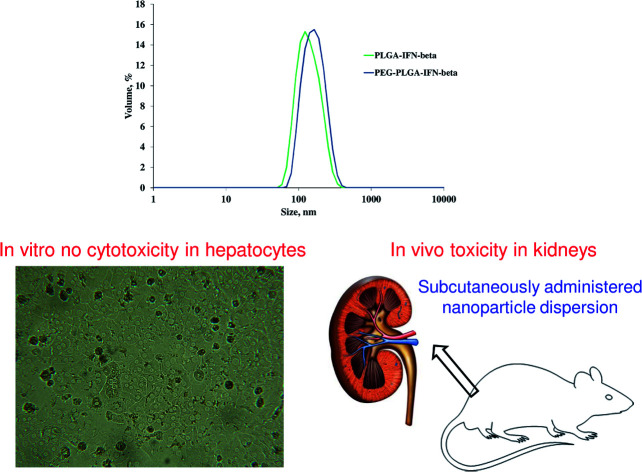

## Introduction

Multiple sclerosis is an autoimmune disease with incidence of genetically prone patients potentially influenced by environmental pathogens.^1^ The US Food and Drug Administration organization approves 8 species of drugs to reduce its symptoms, among which 4 contain interferon-beta (IFN-β). It is effective during the remission of multiple sclerosis and can slow down the physical abnormalities resulting from the disease. It is generally used for a longer time period due to its relatively mild side effects. Interferons are a group of cytokines with antiviral, antiproliferative and immunomodulatory effects. The complete mechanism of IFN-β is still not completely known; however, it is connected to its immunomodulatory capability manifested in the influence on interferon-gamma.^2^ The IFN-β-1a is removed from the circulation primarily by the liver catabolism. The efficiency of interferon therapy is low, since the administered interferon is cleared quickly from the blood circulation after its recognition by the immune system. The half-life of intramuscularly injected IFN-β-1a is 8–10 h.^2,3^ However, it is generally administered intravenously once weekly, it was shown that 3 times injection a week would prolong the beneficial therapeutic effect.^4^ Although three times (44 μg) subcutaneous injection of Rebif® (EMD Serono) caused neutralizing antibody formation in 25% of the patients, while it was observed in only 2% of the cases treated with Avonex® (Biogen) intramuscularly once (30 μg) per week.^5^ Despite the high costs of protein production in mammalian cells compared with recombinant bacterial fermentation, it possesses substantial benefits regarding the synthesized IFN-β.^6^ The controlled release of IFN-β could provide important advantages, such as the decrease of dose and administration frequency, which would reduce the side effects and toxicity, overall the life quality of patients. This reduces the drug cost, immunogenicity and the appearance of neutralizing antibody.^5^

Another promising approach of sustained residence time in blood can be achieved using nanocarriers with controlled release. These nanoparticles can release the drug *via* simple diffusion, dissolution or the degradation of the carrier matrix. The most common preparation techniques are emulsion methods, coacervation/phase separation, antisolvent precipitation, spray or freeze drying, suspension polymerization and spherical agglomeration. Proteins can be microencapsulated most efficiently with polymeric carriers by double emulsion solvent evaporation/diffusion method.^7^ Natural (chitosan, alginic acid, albumins) and synthetic (poly(lactic acid), PLGA, PEG-PLGA) biodegradable and biocompatible polymers are widely used encapsulating materials. Their main tasks are to control the drug liberation and the protection of the active agent from chemical and enzymatic degradation.

Nanoparticulate drug delivery devices can scarcely be found for multiple sclerosis treatment in the scientific literature. However, myelin oligodendrocyte glycoprotein autoantigen and recombinant IL-10 were entrapped by PLGA, and in *in vitro* experiments sustained drug release was observed for both of the agents during the one-month study.^8^ In *in vivo* animal model PLGA-based inverse vaccination delivered subcutaneously was proved to be effective in the treatment of autoimmune diseases. For the treatment of multiple sclerosis by IFN-β-loaded controlled-release composites, microcarrier was synthesized using trimethyl-chitosan, poly(ethylene glycol)dimethacrylate and methacrylic acid in free radical suspension polymerization.^9^ The formed pH-sensitive microparticles were tested after oral administration in rabbits, and 24 h sustained IFN-β was achieved accompanying with higher plasma concentration level compared with the subcutaneously injected IFN-β. In an interesting approach IFN-β-heparin nanocomplexes were prepared and embedded in nanoparticle-releasing methylcellulose and hyaluronan implant formulation injected subcutaneously.^10^ Alginate hydrogels prepared by ionotropic gelation with poly-l-lysine have been investigated as enzymatically degradable stimuli responsive carriers for IFN-β as model drug.^11^

Differently from microencapsulation, PEGylation of IFN-β can also prolong the drug blood life-time.^12–14^ The pharmacokinetics of recombinant human IFN-β-1a in rats was examined,^5^ and unmodified protein represented a half-life of 1 h following intravenous administration, while PEGylated IFN-β-1a suffered much slower elimination half-life (13 h). In another study, PEG-IFN-β-1a showed greater exposure, slower clearance, and decreased distribution volume than unmodified β-1a in Rhesus monkeys.^15^ The drug half-life was significantly higher (20 h) for PEGylated IFN-β-1a than for pure Avonex® (7 h). In a phase I clinical assessment, pharmacokinetic, pharmacodynamic, immune, safety, and tolerability investigations were done.^10^ Although flu like symptoms were reported as adverse effects, the developed material was well tolerated by the volunteers, and the results proved that PEG-conjugated IFN-β can be a potentially effective treatment option for patients with relapsing multiple sclerosis. Finally, cell-penetrating peptides enhanced the nasal mucosal absorption of IFN-β substantially.^16^

In our recent studies protein microencapsulation have been optimized by PLGA and PEG-PLGA copolymers,^17–19^ and IFN-α and PEGylated IFN-α were embedded efficiently in the two carriers, respectively, and they displayed sustained IFN release in human blood plasma.^20^ To our knowledge, nanoparticulate drug delivery systems containing IFN-β-1a have not been developed and tested, yet. Moreover, *in vivo* toxicity of nanoparticles is also scarcely studied. Most of the biocompatibility examinations are limited to the cytotoxicity assays which do not provide reliable information on the effects of nanomedicines in the living organisms. In the present work, we aimed at preparation and investigation of IFN-β-1a-loaded PLGA and PEG-PLGA nanoparticles that might be suitable devices for sustaining the drug release, thus, prolonging its positive effect. The particle preparation process was optimized using bovine serum albumin (BSA) model protein with 4 different types of PLGA and 2 species of PEG-PLGA. After optimization of the preparation process, the size, morphology, encapsulation efficiency and biorelevant *in vitro* release of the selected IFN-β-loaded nanoparticles were studied. Cytotoxicity, cellular uptake and *in vivo* toxicity of the nanoparticles were also evaluated.

## Experimental

### Material

The model drug, bovine serum albumin (BSA) was a kind gift from Trigon Biotechnological Plc. (Hungary). The poly(lactic-*co*-glycolic acid) (PLGA) polymers, Resomer RG 502 H, Resomer 0254 RG: 50:50 H, Resomer RG 504 H and Resomer RG 752 H and PEGylated-PLGA (PEG-PLGA) polymers: Resomer RGP d 5055 (PEG content: 3–7% (m m^−1^)) and Resomer RGP t 50106 (PEG content: 10% (m m^−1^)) were produced by Evonik (Germany). Dichloromethane (DCM), polyvinyl alcohol (PVA, *M*_w_ = 30.000–70.000), *N*-ethyl-*N*′-(3-dimethyl-aminopropyl)-carbodiimide (EDC) crosslinking agent, *N*-hydroxysuccinimide (NHS) stabilizer, phosphate buffered saline tablets (PBS) and ethylene glycol-bis(β-aminoethyl ether)-*N*,*N*,*N*′,*N*′-tetraacetic acid (EGTA) were provided by Sigma Aldrich. The rat IFN-β-1a and corresponding ELISA kit were purchased from Fine Biotech (China). Cyanine-5-amine (Cy5) was obtained from Lumiprobe (Germany). The micro-BCA (bicinchoninic acid) protein assay kit was purchased from Pierce Biotechnology, Inc. (USA).

### Nanoparticle preparation by double emulsion solvent evaporation method

**Scheme 1:**
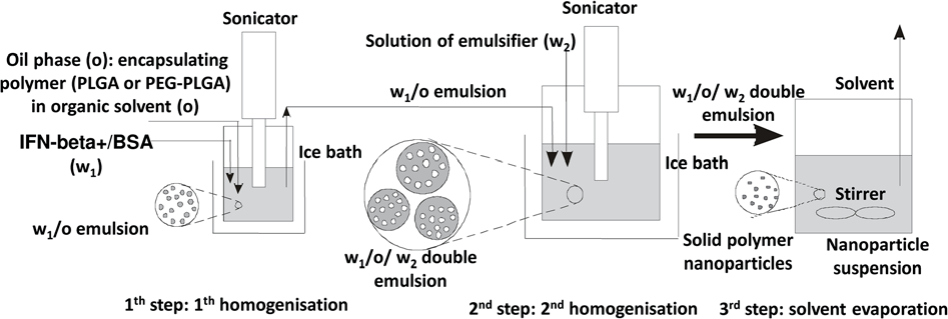
Double emulsion solvent evaporation method.

BSA model protein and IFN-β-1a were encapsulated by water-in-oil-in-water (w_1_/o/w_2_) double emulsion solvent evaporation method^20^ into PLGA and PEG-PLGA nanoparticles (Scheme 1). 0.125–0.50 mg BSA and 1.33 ng to 12.5 μg lyophilised IFN-β-1a dissolved in 0.05–0.2 ml MilliQ water (w_1_) was added to 5.0–20.0 mg PLGA (Resomer RG 502 H, Resomer 0254 RG: 50:50 H, Resomer RG 504 H or Resomer RG 752 H) or PEG-PLGA (Resomer RGP d 5055 or Resomer RGP t 50106) polymer solution in 0.5–2.0 ml dichloromethane (o). The inner aqueous phase (w_1_) was emulsified in the polymeric organic solution (o) using a sonicator (Sonics Vibra Cell VCX 130, 130 W) for 30 seconds at an amplitude of 30%. This primary w_1_/o emulsion was mixed with the 2–8 ml secondary aqueous phase (w_2_) containing 1–2% w/v PVA emulsifier using sonicator for 1–3 min at an amplitude of 70–80%. The formed double emulsion (w_1_/o/w_2_) was magnetically stirred 70–120 min at 25 °C, 500 rpm to evaporate dichloromethane. Blank nanoparticles were prepared by the same method without the active agent as follows: 60 μg BSA dissolved in 50 μl MilliQ water was sonicated with 0.5 ml DCM containing 10 mg Resomer RG 752 H or Resomer RGP t 50106 for 20 s at an amplitude of 30%. The w_1_/o emulsion was added to 1.5% w/v PVA aqueous solution (w_2_) and sonicated for 1–3 min at an amplitude of 70–80%. The formed double emulsion (w_1_/o/w_2_) was magnetically stirred 60 min at 25 °C, 500 rpm to evaporate the solvent.

In another series of samples 12.5 μg lyophilised IFN-β-1a was dissolved in 0.1 ml MilliQ water (w_1_), and 5.0 mg PLGA or PEG-PLGA polymer was dissolved in 0.5 ml dichloromethane (o). The inner aqueous phase (w_1_) was emulsified in the polymeric organic solution (o) using a sonicator (Sonics Vibra Cell VCX 130, 130 W) for 30 seconds at an amplitude of 30%. This primary w_1_/o emulsion was mixed with the 2–8 ml secondary aqueous phase (w_2_) containing 1–2% w/v PVA emulsifier using sonicator for 1 min at an amplitude of 70–80%. The formed double emulsion (w_1_/o/w_2_) was magnetically stirred 70–120 min at 25 °C, 500 rpm to evaporate dichloromethane. After evaporation of the solvent the nanoparticles were recovered by ultracentrifugation (Beckman Optima Max-E ultracentrifuge, Beckman Coulter Inc.) at 40 000×*g* for 10 min. The nanoparticles were washed by resuspension in 2 ml Milli-Q water and centrifuged again. The washing step of IFN-β nanoparticles was repeated twice.

### Nanoparticle preparation by nanoprecipitation method

To prepare blank nanoparticles without BSA, the nanoprecipitation method was utilized.^21^ 5 mg of Resomer 752 H or Resomer RGP t 50106 was dissolved in 0.5 ml acetone under magnetic stirring. A water phase with a volume of 2.0 to 4.0 ml was composed of an aqueous solution (1.0% w/v) of the emulsifying agent PVA. Afterwards, the organic solvent was evaporated over a time period of 12 h at room temperature and 1 bar under constant stirring. The nanoparticles were centrifuged (Eppendorf 5424 R, Hamburg, Germany) at 20 000 rpm for 25 min, washed twice and redispersed in equal volume of MilliQ water.

### Particle size and zeta potential measurements

The size distribution of the nanoparticles was determined by Zetasizer Nano ZS (Malvern Instruments, Malvern, UK) operated upon dynamic light scattering (DLS). The particles were characterized by the volume mean diameter and PdI (polydispersity index). The zeta potential is a characteristic feature of magnitude of the interaction between colloidal particles, and its measurement is commonly used to assess the stability of colloidal systems. The Zetasizer Nano ZS calculates the zeta potential by analysing the electrophoretic mobility and then applying the Henry equation. The electrophoretic mobility is obtained by electrophoresis experiment and velocity measurement using Laser Doppler Velocimetry.

### Morphology of the nanoparticles

The morphology of nanoparticles was imaged with FEI Apreo scanning electron microscope (SEM, Thermofisher, USA) at 20 kV after dropping nanoparticle suspension in MilliQ water onto grid, and drying under room temperature.

### BSA and IFN-β determination

The entrapped BSA was determined indirectly analysing the supernatant after centrifugation of the model drug-loaded nanoparticles by UV/VIS spectrometry using micro BCA protein assay kit at the wavelength of 562 nm.

The encapsulation efficiency and the *in vitro* released amount of IFN-β were determined by enzyme linked immunosorbent assay (ELISA) specific to human and rat IFN-β-1a. The test principle is sandwich enzyme immunoassay. Biotin-conjugated antibody and avidin coupled to horseradish peroxidase form a sandwich which binds to 3,3′,5,5′-tetramethylbenzidine substrate. The IFN-β is complexed with the biotin-conjugated antibody and enzyme-conjugated avidin exhibit the colour change which is measured spectrophotometrically at a wavelength of 450 ± 10 nm with a multiplate reader (Robonik Readwell Touch, India). IFN-β concentration was calculated using a calibration curve in the range of 0–500 pg ml^−1^. The encapsulated mass of IFN-β was measured indirectly in the supernatant after the microencapsulation.

### *In vitro* BSA and IFN-β release

For the investigation of IFN-β decomposition kinetics 52.3 μg ml^−1^ IFN-β solution was prepared in PBS, and the protein concentration was measured for 4 days using the mBCA assay. The absorbance due to the colour reaction was spectrophotometrically analysed at 562 nm in predetermined times.

The prepared BSA-loaded nanoparticles were washed and redispersed in PBS containing 0.03% w/v sodium azide to get a nanoparticle concentration 5–6 mg ml^−1^. The washed IFN-β-loaded nanoparticles were suspended in human blood plasma (1 mg ml^−1^). The release medium was replaced after sampling. The samples were mixed by a rotating mixer (Bio RS-24 mini-Rotator, Biosan, Latvia) with vertical rotation at 30 rpm, and incubated at 37 °C in a G24 Environmental Incubator Shaker (New Brunswick Scientific, USA). At predetermined intervals, 0.5 ml of each sample was ultracentrifuged (Beckman Optima Max-E) for 10 min at 40 000×*g*. The concentration of released IFN-β was determined using ELISA. The BSA concentration was followed by mBCA assay in the supernatant after removal of the nanoparticles by centrifugation, and the IFN-β was analysed in the removed supernatants by ELISA using a multiplate reader.

### Fluorescent dye conjugation

1 ml nanoparticle suspension (2.0 mg ml^−1^) was centrifuged and washed with MilliQ water, and redispersed in 0.5 ml PBS (pH 7.4), and mixed with 0.1 ml PBS (pH 7.4) involving 50× molar excess of EDC and NHS, related to the encapsulating polymer concentration, and incubated for 60 min at 25 °C, centrifuged and washed twice with MilliQ water, and resuspended in 1.0 ml PBS (pH 7.4). 0.02 ml PBS (pH 7.4) solution containing 0.5 mg ml^−1^ Cyanine 5 amine fluorescent dye was added to the prepared carbodiimide activated suspension of nanoparticles, and incubated for 1 h at 25 °C. Then, the dispersion was centrifuged, washed three times and redispersed in 1 ml PBS for the cellular uptake and cytotoxicity experiments.

### Hepatocyte isolation

Primary hepatocytes were prepared from male Wistar rats (Toxi-Coop Toxicological Research Center Ltd., Budapest, Hungary). The liver cells were isolated using collagenase perfusion method.^22^ The liver tissues were perfused through the portal vein with Ca^2+^-free medium (Earle's balanced salt solution) containing EGTA (0.5 mM) and then with the same medium without EGTA, finally with the perfusate containing collagenase (Type IV, 0.25 mg ml^−1^) and Ca^2+^ at physiological concentration (2 mM). The perfusion was done at pH 7.4 and at 37 °C. Softened liver was gently minced and suspended in ice-cold hepatocyte dispersal buffer. Hepatocytes were isolated by low-speed centrifugation (50×*g*), were washed three times and suspended in cell culture medium.^23^

### Cellular uptake and cytotoxicity study

2 × 10^5^ hepatocytes per ml were incubated with 100 μg Cy5-labelled nanoparticles at 37 °C in a humid atmosphere containing 5% CO_2_. At various time points (0, 10, 20, 30, 60, 120, 180, 240 min), the incubation mixtures were sampled, and the medium was removed by low-speed centrifugation. The cellular uptake of nanoparticles was evaluated by fluorescent activated cell sortiment (FACS, Beckman Coulter, Cytoflex S). Hepatocyte viability was determined by trypan blue exclusion.^24^ The experiments were triplicated using cells from 3 different animals.

### *In vivo* toxicity study

Wistar male rats, Crl:(WI)BR (*n* = 5/group) in acceptable health condition were selected for the test. Each animal was examined 24 h before starting the test. All animals were sorted according to body weight and grouped according to weight ranges. There were an equal number of animals from each weight group in each of the experimental groups during the randomisation. The animals received ssniff® SM R/M-Z + H complete diet produced by ssniff Spezialdiäten GmbH (Soest, Germany) ad libitum. The food was periodically analysed and was considered not to contain any contaminants that could reasonably be expected to affect the purpose or integrity of the study. Detailed clinical observations were made on all animals daily. Observation was performed on the skin, fur, eyes and mucous membranes, autonomic activity (lacrimation, piloerection, pupil size, respiratory pattern, occurrence of secretions and excretions), circulatory and central nervous system, somatomotor activity and behaviour pattern, changes in gait, posture and response to handling. Special attention was directed towards the observation of tremors, convulsions, salivation, diarrhoea, lethargy, sleep and coma.

The body weights were recorded on day 1 (just before the treatment) and on day 8, 15 with a precision of 1 g.

The positive control containing IFN-β solution, nanoparticle controls containing blank nanoparticles and the IFN-β-loaded nanoparticles were injected into the neck region of the animals subcutaneously once in the beginning of the study (day 1). 0.2 ml (2.5 mg nanoparticle per ml) was injected to each of the treated animals. The IFN-β dose in the drug-solution- and nanoparticle-treated rats was 0.12 μg per animal. The absolute control group did not get any treatment.

On day 15, animals were over-anesthetized with Isofluran CP®. All animals were subjected to gross pathology in the end of the study. After examination of the external appearance, the cranial, thoracic and abdominal cavities were opened, and the major organs (live, heart, lung, kidney and spleen) were surgically removed and investigated. All gross pathological changes were recorded for each animal.

The study was conducted according to the National Research Council Guide for the Care and Use of Laboratory Animals (Inst. Lab. Anim. Res., *Comm. Life Sci.*, Natl. Acad. Press, Washington, D.C., 8th edn, 2011) and in compliance with the principles of the Hungarian Act 2011 CLVIII (modification of Hungarian Act 1998 XXVIII) and Government Decree 40/2013 regulating animal protection. The study was permitted by Animal Care and Use Committee (IACUC) of Toxi-Coop Ltd. (Budapest, Hungary), then, this toxicological company did the *in vivo* investigations.

### Statistics

All data are expressed as the mean value ± standard deviation (SD), which were calculated and plotted using Microsoft Excel (Microsoft, Redmond, WA). All nanoparticle formulations were produced in three batches (*n* = 3). During *in vivo* tests 5 animals per group were studied.

## Results and discussion

### Optimisation of particle preparation using BSA model protein

Due to the high cost of IFN-β, the process optimization was carried out by a model protein. Previously we investigated the microencapsulation of human interferon-alpha by PLGA and PEG-PLGA,^20^ and in the process optimisation we found that the entrapped BSA and interferon-alpha showed similar physical and chemical properties; thus, we hypothesised that BSA might be an appropriate model protein for IFN-β microencapsulation as well. Four types of PLGA and two types of PEG-PLGA copolymers were applied as encapsulating materials to find the most suitable conditions for IFN-β-1a embedment. The four PLGA and two PEG-PLGA copolymers represented a wide viscosity range of the available materials. By increasing the sonication duration and the ratio of the outer water phase and the organic phase, desirably small nanoparticles could be formed also with the highly viscous polymers Table S1 (in ESI†) shows the optimized process parameters and the results concerning size and encapsulation efficiency. The low polydispersity indices also indicated monomodal and narrow size distribution of each of the nanoparticles. The optimal parameters of the nanoparticles can be defined as the lowest size and highest encapsulation efficiency, thus, Resomer RG 752 H PLGA and Resomer RGP t 50106 PEG-PLGA copolymers were selected for the preparation of IFN-β-1a nanocarriers. Zeta potential values of the nanoparticles (17.7 mV and 18.8 mV for PLGA-IFN and PEG-PLGA-IFN, respectively) might not indicate stable suspension of the nanoparticles, nevertheless, their agglomeration was not observed, which must be due to the steric repulsion resulted from the PVA adsorption that impedes the aggregation. These values are in accordance with the zeta potential result of −15 mV found by Huang *et al.*^21^ using 10 mg ml^−1^ polymer concentration similar to our optimal experiments. They also stated that the zeta potential depended on the polymer concentration.

### IFN β-1a-loaded PLGA and PEG-PLGA nanoparticles

**Fig. 1:**
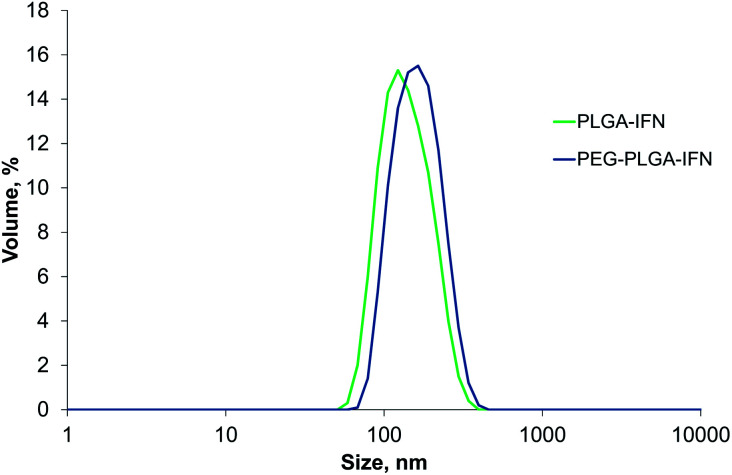
Size distribution by volume of IFN-β-1a-loaded PLGA (PLGA-IFN) and PEG-PLGA nanoparticles (PEG-PLGA-IFN).

With the optimized manufacturing parameters (see Table S1†), IFN-β-1a could be also encapsulated with correspondingly small size as with BSA model protein using the selected encapsulating polymers. The obtained volume mean diameters were 150 ± 10.4 nm (PdI 0.081 ± 0.030) and 165 ± 4.0 nm (PdI 0.093 ± 0.010) for Resomer RG 752 H and Resomer RGP d 5055, respectively. Fig. 1 shows the size distributions of the IFN-β-1a containing nanoparticles.

The encapsulation efficiency of nanoparticles prepared by PLGA was similarly high (96.2%) to that found for BSA encapsulation, while entrapment efficiency was substantially improved for IFN-β-1a using PEG-PLGA (95.9%) compared to BSA encapsulation with the same polymer.

**Fig. 2:**
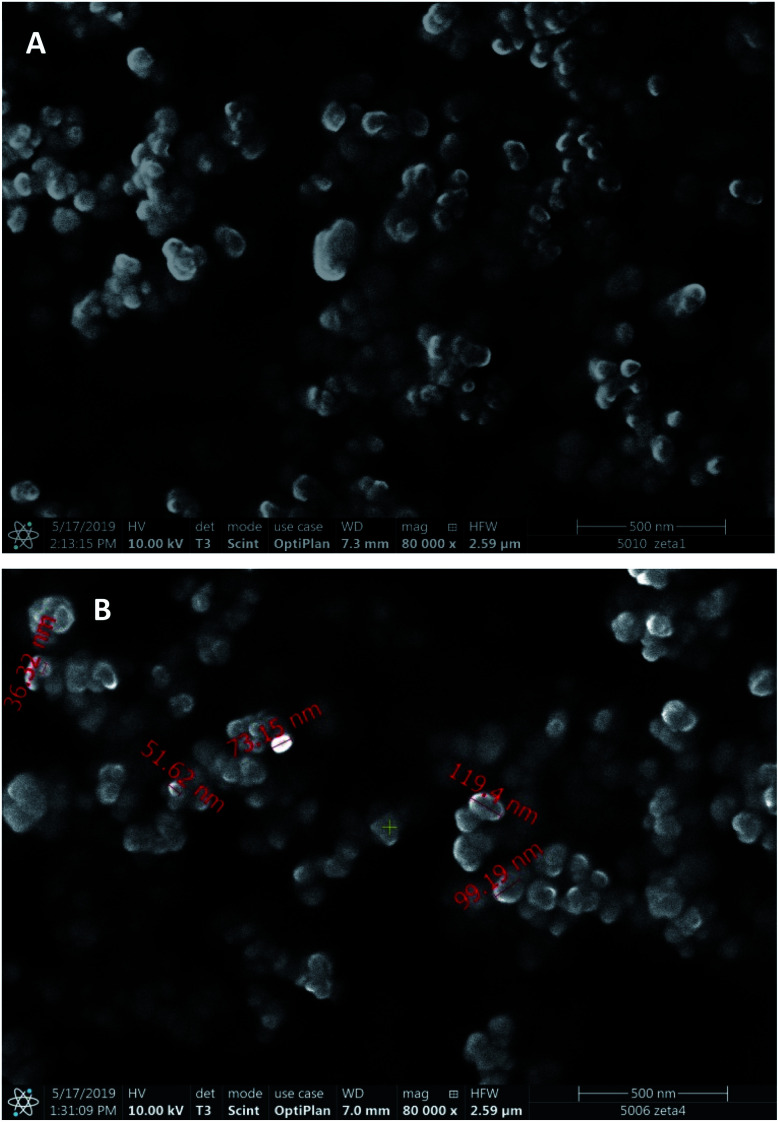
Scanning electron microscopic images of PLGA- (A) and PEG-PLGA-IFN-β-1a (B) nanoparticles.

Scanning electron microscopic images (Fig. 2) display spherical nanoparticles with smaller size (50–120 nm) than that followed from the DLS measurements, which can be explained by the strong PVA adsorption and the difference of hydrodynamic and dry size.

### BSA and IFN-β release kinetics

**Fig. 3:**
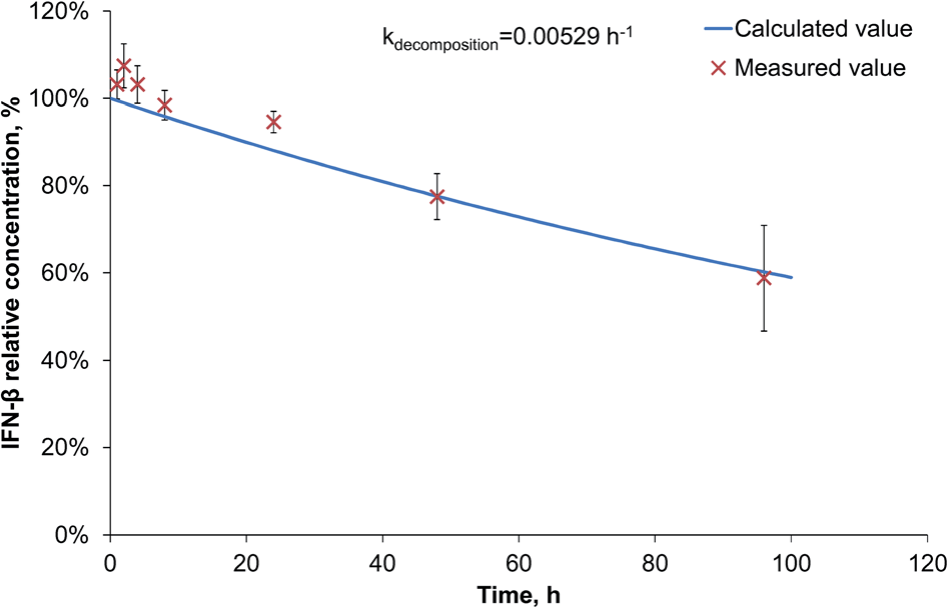
IFN-β degradation kinetics.

Since during the preparation optimization, BSA was found to be a useful model protein, we also investigated its release kinetics from the nanoparticles in order to make a comparison with IFN-β. For the release kinetics analysis, the ModelMaker® software was used, and the data could be approached by first-order kinetics for both of the IFN-β decomposition and the release of IFN-β and BSA proteins. The modelled release can be described by two-phase initial burst and saturation similarly to that drawn by Xu *et al.*^25^ The IFN-β degradation was found to be relatively slow (Fig. 3), characterised by a kinetic constant 0.00529 h^−1^.

The release study was conducted for 2 weeks, and the model calculations indicated complete release of the model drug from PLGA nanoparticles till the end of the study; however, due to its degradation, the relative protein concentration varied in the range of 27–57% reaching a maximum on the 4th day (Fig. S1 in ESI†).

**Fig. 4:**
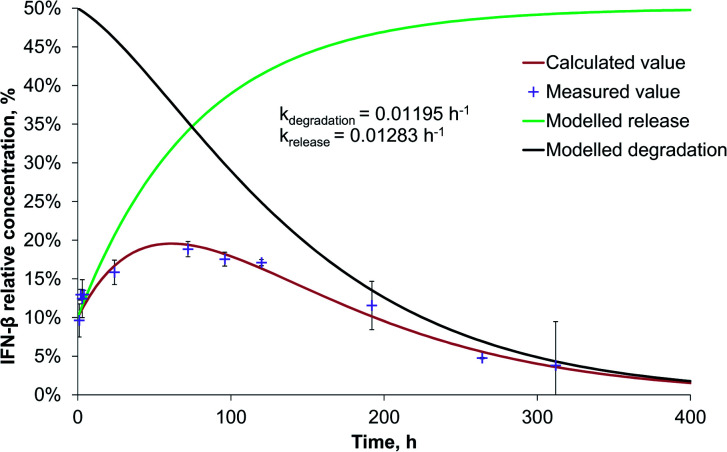
IFN-β release and degradation kinetics of PLGA-IFN-β nanoparticles.

The release rate of IFN-β was very similar to that of BSA from PLGA nanoparticles, though, because of the significantly faster degradation of IFN-β, its relative concentration reached its maximum (20%) after 56 h, and at the end of the two-week study, it became 4% related to the initial IFN-β concentration (Fig. 4).

Interestingly, the PEG-PLGA nanoparticles released the BSA model protein and IFN-β substantially slower than the nanoparticles formed with PLGA encapsulating polymer (Fig. S2† and 5).

**Fig. 5:**
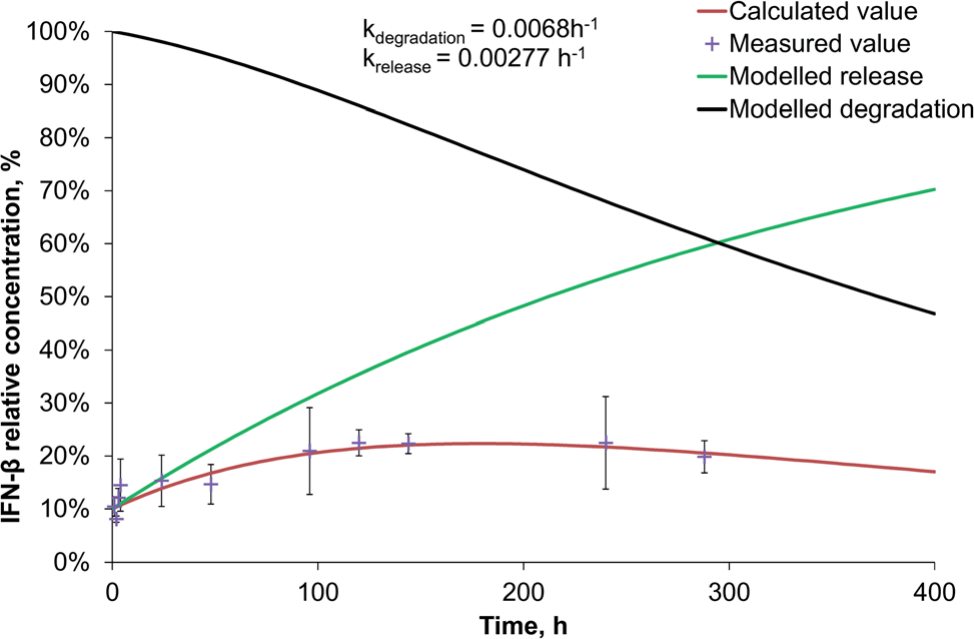
IFN-β release and degradation kinetics of PEG-PLGA-IFN-β nanoparticles.

The release rate of BSA (0.00207 h^−1^) was very similar to that of IFN-β (0.00277 h^−1^) in PEG-PLGA nanoparticles. Due to the 3 times higher degradation rate of IFN-β related to that of BSA, the maximum relative IFN-β concentration was 23% after 5 days, while BSA had a plateau at 34%, that remained almost constant till the end of the study. The release rate is influenced by numerous parameters of proteins and encapsulating polymers. At the beginning, the protein release was diffusion-controlled through the pores, then a saturation phase followed, finally, the polymer degradation influenced mostly the drug liberation resulting in an acidic microenvironment for the protein. The pH decrease can cause aggregation of the protein.^26^

However, besides IFN-β, several other disease modifying therapies have been approved for the treatment of multiple sclerosis, interferons retain the standard care in the treatment of relapsing-remitting multiple sclerosis.^27^ Most of the new concerning researches focus on the development of new therapeutic agents and pay less attention to the formulation of present drugs in order to improve the therapeutic and to decrease the side effects.^28^ The reason in the case of IFN-β is surely its high cost. Since very limited number of papers are available in IFN-β drug delivery by nano or microparticles, the comparison with literature data is quite difficult. The most promising results in this aspect were published by Kondiah *et al.*^29^ They prepared pH responsive copolymeric trimethyl chitosan-poly(ethylene glycol)-dimethacrylatemethacrylic acid microparticles by free radical suspension polymerisation technique for oral IFN-β controlled delivery. However, for IFN-β, the commercially available and clinically tested products are exclusively intramuscular or subcutaneous formulations.^28^ Nevertheless the pharmacodynamics of IFN-β in multiple sclerosis is complex and still poorly understood.^30^

### Cellular uptake and cytotoxicity

**Fig. 6:**
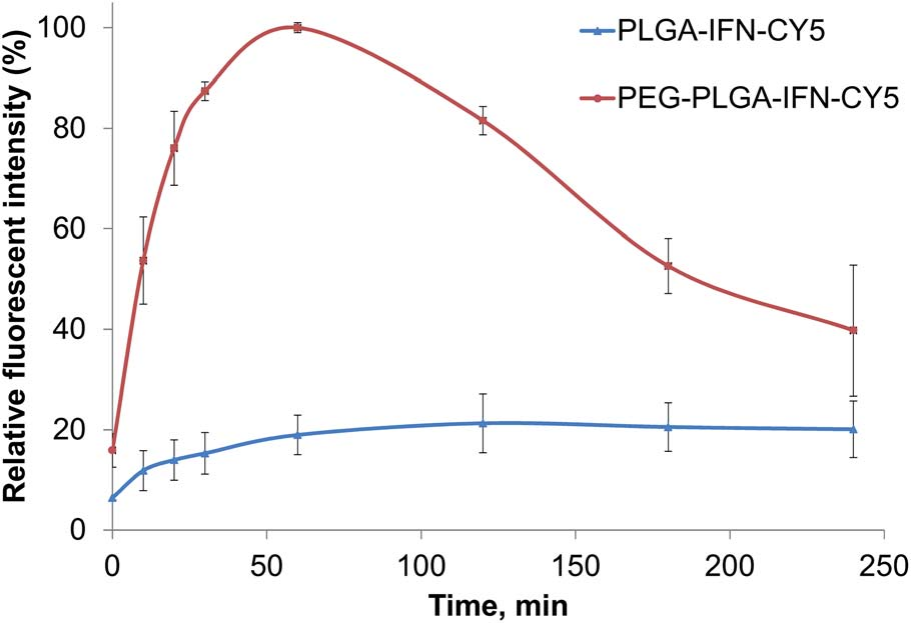
Relative fluorescent intensity of IFN-β-loaded PLGA (PLGA-IFN-CY5) and PEG-PLGA (PEG-PLGA-IFN-CY5) nanoparticles as a function of time.

In cellular uptake studies performed with FACS in hepatocytes, PEG-PLGA containing IFN-β showed a maximum concentration at 60 min, and then the fluorescent intensity reduced (Fig. 6). While the uptake of PLGA containing IFN-β was slower with the maximum concentration between 120 and 180 min. Then the nanoparticles and/or the fluorescent dye were degraded or removed from the cells.

**Fig. 7:**
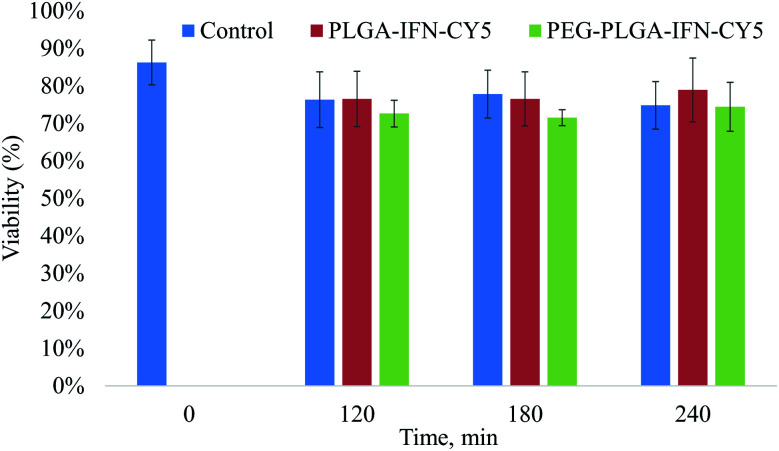
Viability of hepatocytes treated with IFN-β-loaded PLGA (PLGA-IFN-CY5) and PEG-PLGA (PEG-PLGA-IFN-CY5) nanoparticles, and that of untreated cells (control) as a function of time.

In trypan blue exclusion assay, IFN-β nanocarriers composed of PLGA and PEG-PLGA polymers at a concentration of 100 μg ml^−1^ did not indicate cytotoxic effect compared to the nanoparticle-free incubation (control) of isolated rat hepatocytes during the 4 h incubation time (Fig. 7).

### *In vivo* toxicity studies

The animals were symptom-free during the whole 2 week study. There were no notable or statistically significant differences in the mean bodyweight or bodyweight gain of treated male animals with respect to the relevant absolute control during the study period (Table S2†). There were no pathological changes in the animals of absolute (without treatment) and positive control group treated with IFN-β.

In the group treated with dispersions of PEG-PLGA-BSA blank nanoparticles, pale kidneys were found in two animals during the necropsy, while PLGA-BSA blank nanoparticles did not exert toxic signs (Table S3†). Three of the animals treated with PLGA-BSA-IFN nanoparticles suffered from pale kidneys. PEG-PLGA-BSA-IFN nanoparticles evoked pale kidneys in one animal and pyelectasis on both sides in two other animals. There was not any toxic sign in the animals of the absolute control (untreated animals) and the positive control groups (treated with IFN-β solution).

Since both the blank- and the IFN-β-loaded nanoparticles showed toxic impacts, while the IFN-β solution did not induce toxicity, we changed the nanoparticle preparation process in order to avoid the toxic effect of nanoparticles. In the changed methods, BSA was not involved in the preparation process of all of the nanoparticles; furthermore, the blank nanoparticles were formed with nanoprecipitation, and in this way the DCM solvent was substituted by acetone. However, nanoparticles entrapping BSA beside the IFN-β were also investigated in the second round of the *in vivo* study in order to exclude the technical error of the first *in vivo* experiments.

During the new, improved two-week study, the controls and the treated groups did not evoke toxic symptoms in the clinical signs, bodyweight and in bodyweight gain (Table S4†). However, the necropsy showed again pale kidneys and pyelectasis in almost all of the animal groups that obtained blank or drug-loaded nanoparticles, while the animals of absolute control group were symptom free also in necropsy, and IFN-β drug solution did neither exert any toxic effect (Table S5†). In groups treated with dispersions of blank PLGA and PLGA-PEG nanoparticles, pale kidneys and pyelectasis on both sides were found in one animal of each groups, moreover, pale kidneys were observed in one more animal of the group treated with PLGA-PEG nanoparticles. In the group treated with PLGA-BSA-IFN, three animals had pale kidneys, and in two of them pyelectasis on both sides were also noticed. In the group administered with PEG-PLGA-BSA-IFN, two animals possessed pale kidneys. In the groups treated with PLGA-IFN and PEG-PLGA-IFN nanoparticles, pale kidneys were found in two and three animals, respectively. As the SEM images display significantly smaller sizes than DLS measurements, probably a part of the particles was below 50 nm which is the limit of renal filtration. In this case, the nanoparticles could cause obstruction in the kidneys. However, the exact reason for the experienced toxicity requires further investigations.

Unfortunately, most of the published nanotoxicity studies have been achieved exclusively in cell cultures so far, consequently there is only very limited number of available information on *in vivo* toxicity of nanoparticles. The promising *in vitro* toxicity data should be verified *in vivo*, since toxicity studies in cell culture can overestimate or underestimate toxicity in living organisms. Oral^31^ and pulmonary^32^ delivery of PLGA nanoparticles did not induce any toxicity in mice; however, the mean size of the studied nanoparticles was 200–300 nm. Paclitaxel- and superparamagnetic iron oxide-loaded PLGA and PEG-PLGA nanoparticles with size >200 nm were injected intravenously into mice.^33^ In that study aspartate aminotransferase and alanine aminotransferase for hepatic function, blood urea nitrogen for kidney function and creatine kinase isoenzyme as cardiac marker levels were analysed, whilst none of them indicated toxic effect of the nanoparticles. However, nanoparticles prepared using monomethoxy(polyethyleneglycol)-poly(d,l-lactic-*co*-glycolic acid)-monomethoxy polymer with average size of 50 nm caused significant immunotoxicity at low or high doses in Wistar rats after intravenous administration, while the same doses of 200 nm nanoparticles did not exert any toxicity.^34^ These results in accordance with our finding suggest that nanoparticle toxicity *in vivo* is highly dependent on the administration route and the particle size.

## Conclusions

BSA model protein was used to optimize the size and encapsulation efficiency of drug-loaded PLGA and PEG-PLGA nanoparticles. Under the optimal conditions, IFN-β was microencapsulated by the selected PLGA and PEG-PLGA carrier polymers with similar size and high encapsulation efficiency as the model protein. The release kinetics of the model and active agents could be described with first-order kinetics. The release rates of BSA and IFN-β were very similar in the PLGA and PEG-PLGA polymers, respectively, nevertheless IFN-β had significantly quicker degradation rate in both of the polymers. Although, the molecular weight of BSA is significantly higher than that of IFN-β, its behaviour in the encapsulation process and release study confirmed that it can be a useful protein to model IFN-β microencapsulation. The IFN-β-loaded nanoparticles were taken up effectively by primary hepatocytes, and they did not have cytotoxic effects *in vitro*. However, the *in vivo* toxicity study showed mild toxic signals of drug-loaded as well as blank PLGA and PEG-PLGA nanoparticles. The reason of the renal toxicity may be related to the renal filtration of the nanoparticles due to their small size; however, further *in vivo* studies are needed to confirm this hypothesis.

## Conflicts of interest

There are no conflicts to declare.

## Supplementary Material

RA-010-C9RA09928J-s001

## References

[cit1] Badawi A. H., Siahaan T. J. (2012). Clin. Immunol..

[cit2] Holliday S. M., Benfield P. (1997). BioDrugs.

[cit3] Khan O. A., Dhib-Jalbut S. S. (1998). Neurology.

[cit4] Wagstaff A. J., Goa K. L. (1998). BioDrugs.

[cit5] Baker D. P., Lin E. Y., Lin K. C., Pellegrini M., Petter R. C., Chen L. L. (2006). et al.. Bioconjugate Chem..

[cit6] Lipiäinen T., Peltoniemi M., Sarkhel S., Yrjönen T., Vuorela H., Urtti A. (2015). et al.. J. Pharm. Sci..

[cit7] Jain R. A. (2000). Biomaterials.

[cit8] Cappellano G., Woldetsadik A. D., Orilieri E., Shivakumar Y., Rizzi M., Carniato F. (2014). et al.. Vaccine.

[cit9] Kondiah P. P. D., Tomar L. K., Tyagi C., Choonara Y. E., Modi G., Du Toit L. C. (2013). et al.. Int. J. Pharm..

[cit10] Beyer S., Xie L., Schmidt M., De Bruin N., Ashtikar M., Rüschenbaum S. (2016). et al.. J. Controlled Release.

[cit11] Tolle C., Riedel J., Mikolai C., Winkel A., Stiesch M., Wirth D. (2018). et al.. Biomolecules.

[cit12] Calabresi P. A., Kieseier B. C., Arnold D. L., Balcer L. J., Boyko A., Pelletier J. (2014). et al.. Lancet Neurol..

[cit13] Seddighzadeh A., Hung S., Selmaj K., Cui Y., Liu S., Sperling B. (2014). et al.. Expert Opin. Drug Delivery.

[cit14] Khan U. T., Tanasescu R., Constantinescu C. (2015). Expert Opin. Biol. Ther..

[cit15] Hu X., Olivier K., Polack E., Crossman M., Zokowski K., Gronke R. S. (2011). et al.. J. Pharmacol. Exp. Ther..

[cit16] Iwase Y., Kamei N., Khafagy E. S., Miyamoto M., Takeda-Morishita M. (2016). Int. J. Pharm..

[cit17] Shubhra Q. T. H., Kardos A. F., Feczkó T., Mackova H., Horák D., Tóth J. (2014). et al.. J. Microencapsulation.

[cit18] Shubhra Q. T. H., Feczkó T., Kardos A. F., Tóth J., Mackova H., Horak D. (2014). et al.. J. Microencapsulation.

[cit19] Shubhra Q. T. H., Tóth J., Gyenis J., Feczkó T. (2014). Colloids Surf., B.

[cit20] Feczkó T., Fodor-Kardos A., Sivakumaran M., Shubhra Q. T. H. (2016). Nanomedicine.

[cit21] Huang W., Zhang C. (2018). Biotechnol. J..

[cit22] Bayliss K. M. and Skett P., Human Cell Culture Protocols, in Human Cell Culture Protocols, ed. G. E. Jones, Humana Press, Totowa, 1996, pp. 369–390

[cit23] Ferrini J. B. , Ourlin J., Pichard L., Fabre G. and Maurel P., Human hepatocyte culture, in Cytochrome P450 protocols, ed. I. R. Phillips and E. A. Shephard, Humana Press, Totowa, 1998, pp. 341–352 10.1385/0-89603-519-0:341 14577243

[cit24] Berry M. N. , Edwards A. M., Barritt G. J., Grivell M. B., Halls H. J., Gannon B. J., et al., Initial determination of cell quality. Isolated Hepatocytes, Elsevier, Amsterdam, 1991, pp. 45–47

[cit25] Xu Y., Kim C. S., Saylor D. M., Koo D. J. (2017). J. Biomed. Mater. Res., Part B.

[cit26] Giteau A., Venier-Julienne M. C., Aubert-Pouëssel A., Benoit J. P. (2008). Int. J. Pharm..

[cit27] Madsen C. (2017). Brain Behav..

[cit28] Saleem S., Anwar A., Fayyaz M., Anwer F., Anwar F. (2019). Cureus.

[cit29] Kondiah P. P. D., Choonara Y. E., Tomar L. K., Tyagi C., Kumar P., du Toit L. C. (2017). et al.. AAPS PharmSciTech.

[cit30] Roselli F., Chandrasekar A., Morganti-Kossmann M. C. (2018). Front. Neurol..

[cit31] Semete B., Booysen L., Lemmer Y., Kalombo L., Katata L., Verschoor J. (2010). et al.. Nanomedicine.

[cit32] Aragao-Santiago L., Hillaireau H., Grabowski N., Mura S., Nascimento T. L., Dufort S. (2016). et al.. Nanotoxicology.

[cit33] Ganipineni L. P., Ucakar B., Joudiou N., Bianco J., Préat V., Bastiancich C. (2018). et al.. Int. J. Nanomed..

[cit34] Liao L., Zhang M., Liu H., Zhang X., Xie Z., Zhang Z. (2014). et al.. Nanotechnology.

