# Supplementary material for: Sustained in vitro interferon-beta release and in vivo toxicity of PLGA and PEG-PLGA nanoparticles
Source: RSC Adv. 2020 Apr 22;10(27):15893–900. doi: 10.1039/c9ra09928j (PMC9052435; doi:10.1039/c9ra09928j)
Supplement: RA-010-C9RA09928J-s001 [file RA-010-C9RA09928J-s001.pdf]

Electronic Supplementary Material (ESI) for RSC Advances.
This journal is © The Royal Society of Chemistry 2020

Supplementary material

Sustained in vitro interferon-beta release and in vivo toxicity of PLGA and PEG-PLGA nanocomposites

Andrea Fodor-Kardos[a,b], Ádám Ferenc Kiss[c], Katalin Monostory[c], Tivadar Feczkó[a,b*]

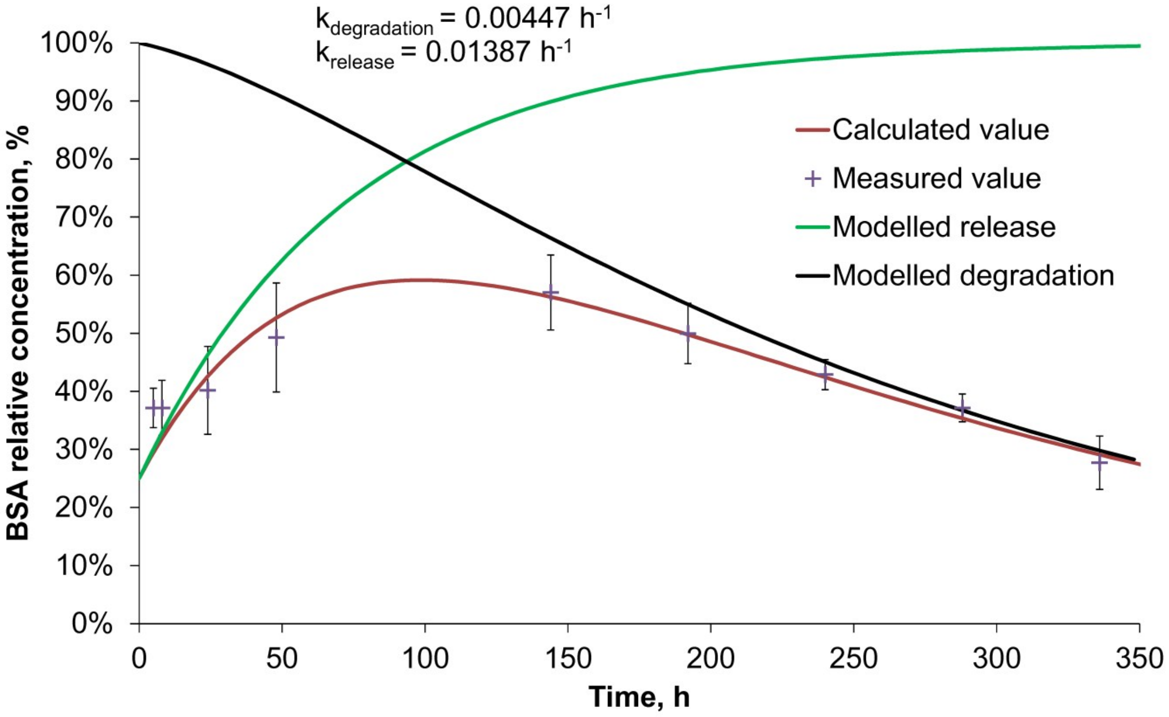

Figure S1. BSA release and degradation kinetics of PLGA-BSA nanoparticles.

[a.] Institute of Materials and Environmental Chemistry, Research Centre for Natural Sciences, Magyar Tudósok Körútja 2., H-1117, Budapest, Hungary
[b.] Research Institute of Biomolecular and Chemical Engineering, University of Pannonia, Egyetem u. 10., H-8200, Veszprém, Hungary
[c.] Institute of Enzymology, Research Centre for Natural Sciences, Magyar Tudósok Körútja 2., H-1117, Budapest, Hungary
[d.] * Correspondence: tivadar.feczko@gmail.com; Tel.: +36-88-624000/3508

Electronic Supplementary Information (ESI) available: [details of any supplementary information available should be included here]. See DOI: 10.1039/x0xx00000x

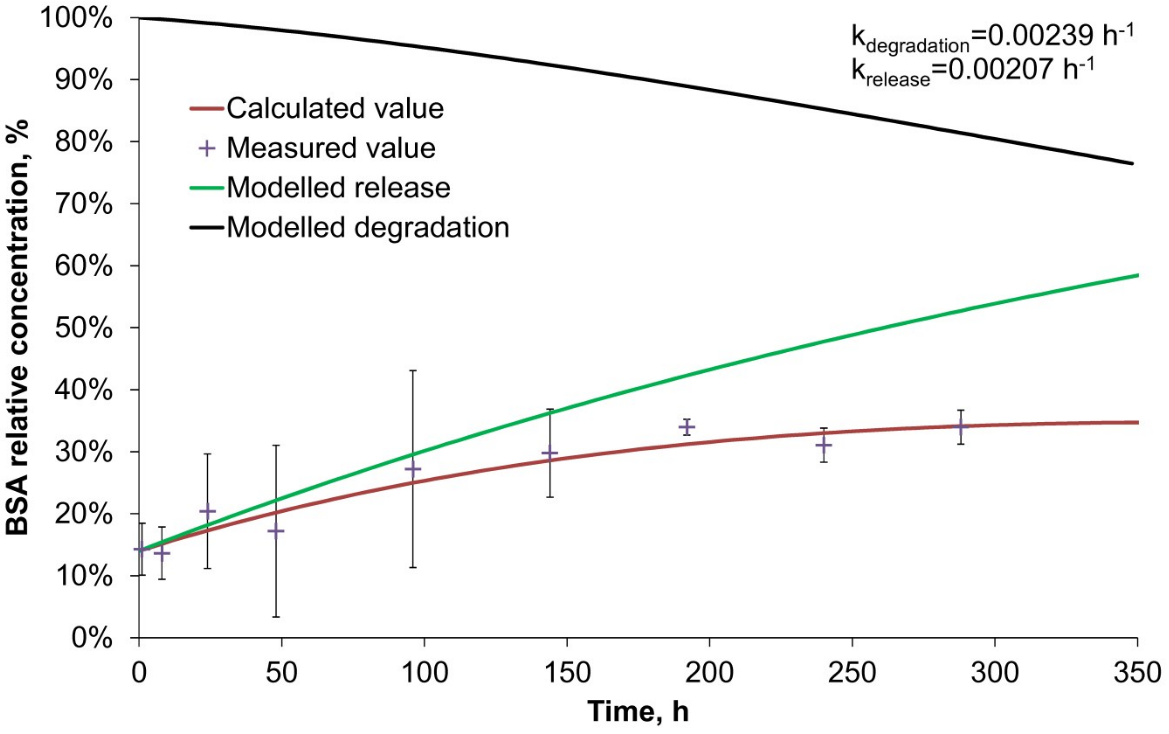

Figure S2. BSA release and degradation kinetics of PEG-PLGA-BSA nanoparticles.

Table S1. Processing parameters, volume mean diameter (D[4,3]), polydispersity index (PdI) and encapsulation efficiency (EE) of BSA-loaded PLGA and PEG-PLGA nanoparticles

Polymer	polymer inherent viscosity (dL/g)	w2/o volume ratio	Probe diameter, mm	sonication time (s)	D[4,3] (nm)	PdI	EE (%)
**PLGA 752H**	0.14-0.22	1:4	6	90	144	0.088	98.7
**PLGA 502H**	0.16-0.24	1:2	3	180	173	0.070	86.6
**PLGA 0254**	0.32	1:3	3	180	162	0.081	87.0
**PLGA 504H**	0.45-0.60	1:6	6	110	168	0.078	87.3
**PEG-PLGA_5050**	0.67	1:6	6	140	174	0.078	69.0
**PEG-PLGA 50106**	0.93	1:4	6	90	157	0.061	78.0

Table S2. Individual body weight and weight gain in the *in vivo* study. * no data

**Group 1, absolute control** Animal No.	Days 1	8	15	Mean body weight (g)	Weight gain (g)
1	261	295	320	292.0	59
2	255	291	317	287.7	62
3	258	290	318	288.7	60
4	274	296	319	296.3	45
5	288	313	338	313.0	50
Group mean	267.2	297.0	322.4	295.5	55.2
**Group 2. IFN-β control** Animal No.	Days 1	8	15	Mean body weight (g)	Weight gain (g)
6	270	309	340	306.3	70
7	264	293	325	294.0	61
8	274	306	330	303.3	56
9	281	313	342	312.0	61

10	276	310	*	*	*
Group mean	273.0	306.2	334.25	303.9	62
**Group 3, PLGA-BSA blank nanoparticles**, animal No.	Days			Mean body weight (g)	Weight gain (g)
	1	8	15		
11	278	310	341	309.7	63
12	280	322	347	316.3	67
13	264	305	332	300.3	68
14	262	299	335	298.7	73
15	260	275	310	281.7	50
Group mean	278	310	341	301.3	64.2
**Group 4. PEG-PLGA-BSA blank nanoparticles** Animal No.	Days			Mean body weight (g)	Weight gain (g)
	1	8	15		
16	273	310	339	307.3	66
17	284	317	*	*	*
18	275	311	336	307.3	61
19	267	302	330	299.7	63
20	258	265	297	273.3	39
Group mean	271.4	301.0	325.5	296.9	57.3
**Group 5, PLGA-BSA-IFN nanoparticles**, animal No.	Days			Mean body weight (g)	Weight gain (g)
	1	8	15		
21	257	298	333	296.0	76
22	273	312	345	310.0	72
23	267	290	319	292.0	52
24	269	303	328	300.0	59
25	278	300	336	304.7	58
Group mean	268.8	300.6	332.2	300.5	63.4
**Group 6. PEG-PLGA-BSA-IFN nanoparticles** Animal No.	Days			Mean body weight (g)	Weight gain (g)
	1	8	15		
26	273	320	350	314.3	77
27	270	306	339	305.0	69
28	273	292	328	297.7	55
29	267	289	315	290.3	48
30	265	307	329	300.3	64
Group mean	273	320	350	301.5	62.6

Table S3. Observations of necropsy following *in vivo* study.

**Group 1, absolute control**	
Animal No.:	**Observations**
1	No macroscopic findings
2	No macroscopic findings
3	No macroscopic findings
4	No macroscopic findings
5	No macroscopic findings
**Group 2, IFN-β control**	
Animal No.:	**Observations**
6	No macroscopic findings
7	No macroscopic findings
8	No macroscopic findings
9	No macroscopic findings
10	No macroscopic findings
**Group 3, PLGA-BSA blank nanoparticles**	
Animal No.:	**Observations**
11	No macroscopic findings
12	No macroscopic findings
13	No macroscopic findings
14	No macroscopic findings
15	No macroscopic findings
**Group 4, PEG-PLGA-BSA blank nanoparticles**	
Animal No.:	**Observations**
16	No macroscopic findings
17	No macroscopic findings
18	No macroscopic findings
19	Pale kidneys
20	Pale kidneys
**Group 5, PLGA-BSA-IFN nanoparticles**	
Animal No.:	**Observations**
21	Pale kidneys
22	Pale kidneys
23	Pale kidneys
24	No macroscopic findings
25	No macroscopic findings
**Group 6, PEG-PLGA-BSA-IFN nanoparticles**	
Animal No.:	**Observations**
26	Pale kidneys
27	Pyelectasis on both sides
28	No macroscopic findings
29	No macroscopic findings
30	Pyelectasis on both sides

Table S4. Individual body weight and weight gain in the second *in vivo* study.

**Group 1, absolute control** Animal No.	Days 1	8	15	Mean body weight (g)	Weight gain (g)
1	293	316	350	319.7	57
2	300	326	349	325.0	49
3	309	332	358	333.0	49
4	318	364	413	365.0	95
5	303	327	328	319.3	25
Group mean	304.6	333.0	359.6	332.4	55.0
**Group 2. IFN-β control** Animal No.	Days 1	8	15	Mean body weight (g)	Weight gain (g)
6	294	319	352	321.7	58
7	299	326	363	329.3	64
8	318	348	382	349.3	64
9	309	335	367	337.0	58
10	307	336	362	335.0	55
Group mean	305.4	332.8	365.2	334.5	59.8
**Group 3, PLGA blank nanoparticles,** animal No.	Days 1	8	15	Mean body weight (g)	Weight gain (g)
11	294	317	352	321.0	58
12	299	328	350	325.7	51
13	317	349	387	351.0	70
14	309	338	379	342.0	70
15	305	330	361	332.0	56
Group mean	304.8	332.4	365.8	334.3	61.0
**Group 4, PEG-PLGA blank nanoparticles,** animal No.	Days 1	8	15	Mean body weight (g)	Weight gain (g)
16	305	344	375	341.3	70
17	307	329	368	334.7	61
18	316	354	389	353.0	73
19	297	330	367	331.3	70
20	295	323	371	329.7	76
Group mean	304.0	336.0	374.0	338.0	70.0
**Group 5, PLGA-BSA-IFN nanoparticles,** animal No.	Days 1	8	15	Mean body weight (g)	Weight gain (g)
21	296	324	359	326.3	63
22	306	324	358	329.3	52
23	299	325	347	323.7	48
24	316	351	389	352.0	73
25	308	345	382	345.0	74
Group mean	305.0	333.8	367.0	335.3	62.0
**Group 6. PEG-PLGA-BSA-IFN nanoparticles** Animal No.	Days 1	8	15	Mean body weight (g)	Weight gain (g)
26	296	333	372	333.7	76
27	307	339	377	341.0	70
28	305	340	364	336.3	59
29	301	337	374	337.3	73
30	311	348	388	349.0	77
Group mean	304.0	339.4	375.0	339.5	71.0
**Group 7, PLGA-IFN nanoparticles,** animal No.	Days 1	8	15	Mean body weight (g)	Weight gain (g)
31	305	331	354	330.0	49
32	309	336	364	336.3	55
33	298	326	371	331.7	73
34	302	334	365	333.7	63
35	308	333	369	336.7	61
Group mean	304.4	332.0	364.6	333.7	60.2

**Group 8, PEG-PLGA-IFN nanoparticles,** animal No.	Days 1	8	15	Mean body weight (g)	Weight gain (g)
36	302	316	330	316.0	28
37	297	300	338	311.7	41
38	310	343	368	340.3	58
39	302	321	344	322.3	42
40	305	330	356	330.3	51
Group mean	303.2	322.0	347.2	324.1	42.6

Table S5. Observations of necropsy following the second *in vivo* study

**Group 1, absolute control**	
Animal No.:	**Observations**
1	No macroscopic findings
2	No macroscopic findings
3	No macroscopic findings
4	No macroscopic findings
5	No macroscopic findings
**Group 2, IFN-β control**	
Animal No.:	**Observations**
6	No macroscopic findings
7	No macroscopic findings
8	No macroscopic findings
9	No macroscopic findings
10	No macroscopic findings
**Group 3, PLGA blank nanoparticles**	
Animal No.:	**Observations**
11	No macroscopic findings
12	No macroscopic findings
13	Pale kidneys, pyelectasis on both sides
14	No macroscopic findings
15	No macroscopic findings
**Group 4, PEG-PLGA blank nanoparticles**	
Animal No.:	**Observations**
16	Pale kidneys
17	No macroscopic findings
18	No macroscopic findings
19	No macroscopic findings
20	Pale kidneys, pyelectasis on both sides
**Group 5, PLGA-BSA-IFN nanoparticles**	
Animal No.:	**Observations**
21	Pale kidneys, pyelectasis on both sides
22	No macroscopic findings
23	Pale kidneys
24	Pale kidneys, pyelectasis on both sides
25	No macroscopic findings
**Group 6, PEG-PLGA-BSA-IFN nanoparticles**	
Animal No.:	**Observations**
26	Pale kidneys
27	No macroscopic findings
28	No macroscopic findings
29	Pale kidneys
30	No macroscopic findings
**Group 7, PLGA-IFN nanoparticles**	
Animal No.:	**Observations**
31	Pale kidneys
32	No macroscopic findings
33	No macroscopic findings
34	Pale kidneys
35	No macroscopic findings

**Group 8, PEG-PLGA-IFN nanoparticles**	
Animal No.:	**Observations**
36	Pale kidneys
37	Pale kidneys
38	Pale kidneys
39	No macroscopic findings
40	No macroscopic findings
